# Evolution of Coronary Microvascular Dysfunction Prevalence over Time and Across Diagnostic Modalities in Patients with ANOCA: A Systematic Review

**DOI:** 10.3390/jcm14030829

**Published:** 2025-01-27

**Authors:** Aurelia Zimmerli, Adil Salihu, Panagiotis Antiochos, Henri Lu, Barbara Pitta Gros, Alexandre Berger, Olivier Muller, David Meier, Stephane Fournier

**Affiliations:** Department of Cardiology, Lausanne University Hospital, University of Lausanne, 1011 Lausanne, Switzerland; aurelia.zimmerli@chuv.ch (A.Z.); adil.salihu@chuv.ch (A.S.); panagiotis.antiochos@chuv.ch (P.A.); henri.lu@chuv.ch (H.L.); barbara.pitta-gros@chuv.ch (B.P.G.); alexandre.berger@chuv.ch (A.B.); olivier.muller@chuv.ch (O.M.)

**Keywords:** coronary microvascular dysfunction, prevalence, ANOCA, diagnostic modalities

## Abstract

**Background:** A considerable number of patients with angina undergo invasive coronary angiography, which might reveal non-obstructive coronary arteries (ANOCA). In this setting, they might have coronary microvascular disease (CMD). Its prevalence significantly varies in the literature. This systematic review aims to document the prevalence of CMD over time according to the diagnostic modalities. **Methods:** A systematic literature review was conducted using PubMed, the Cochrane Library, and Embase, covering publications from inception to 1 May 2024. Among 1471 identified articles, 297 full-text articles were assessed for eligibility. All studies reporting the prevalence of CMD in ANOCA patients based on invasive coronary artery (ICA), positron emission tomography–computed tomography (PET-CT), transthoracic echocardiography (TTE), or cardiac magnetic resonance (CMR) were included. **Results:** The review included 53 studies (published between 1998 and 2024), encompassing a total of 16,602 patients. Of these studies, 23 used ICA, 15 used PET-CT, 8 used TTE, and 7 used CMR. A statistically significant increase in CMD prevalence over time was observed across all diagnostic modalities (*p* < 0.05), except for PET-CT, which showed a consistent and stable prevalence over time. Notably, the prevalence rates from all of the diagnostic methods converged towards the 50% prevalence detected by PET-CT. **Conclusions:** The prevalence of CMD in patients with ANOCA is subject to debate. However, the current data suggest that regardless of the diagnostic method used, the most recent studies tend to converge towards a prevalence value of 50%, which has been consistently reported by PET-CT from the beginning.

## 1. Introduction

A considerable proportion of patients experiencing chest pain referred to a catheterization laboratory for suspected coronary artery disease present with non-obstructive coronary artery [1]. The presence of angina and/or ischemia in patients with non-obstructive coronary arteries represents an entity called ANOCA and/or INOCA, respectively. Although obstructive ischemic heart disease remains the leading cause of mortality worldwide [2], the absence of obstructive coronary artery disease is not without consequence. Indeed, the diagnosis of angina/ischemia with non-obstructive coronary artery (ANOCA/INOCA) is associated with increased mortality and major adverse cardiac events (MACEs) [3] and impaired quality of life, and can lead to repeated coronary angiography [4,5,6]. There are different mechanisms implicated such as myocardial bridging, vasospastic angina (VSA), or coronary microvascular dysfunction (CMD).

While the methods and diagnostic criteria for VSA are well established, the same cannot be said for CMD. VSA is assessed invasively using intracoronary angiography (ICA) with the administration of intracoronary acetylcholine and the diagnosis is confirmed by the presence of an epicardial artery spasm of ≥90%, accompanied by ECG repolarization abnormalities and reproduction of the symptoms presented by the patient.

In contrast, evaluating coronary microcirculation is more complex due to the diversity of available diagnostic tools, which include both invasive and non-invasive methods, as well as a lack of standardized diagnostic criteria. The assessment of coronary microcirculation primarily involves measuring the coronary flow reserve (CFR), defined as the ratio of coronary blood flow during hyperemia to that measured at rest. CFR can be assessed through invasive coronary angiography (ICA) [7], considered as the gold standard, as well as through various non-invasive methods, including Doppler transthoracic echocardiography (TTE), positron emission tomography–computed tomography (PET-CT), and cardiovascular magnetic resonance (CMR). Among non-invasive procedures, PET-CT is considered as the gold standard [8,9,10]. Furthermore, variability in the recommended diagnostic cut-off for CFR, ranging from 2.0 to 2.5, depending on the guidelines, adds complexity to the diagnosis. In addition, one invasive method that also allows for the calculation of diagnostic criteria is the index of microvascular resistance (IMR), which can be elevated in the classical form of structural CMD but can be low in the functional form of CMD.

Indeed, the reported prevalence of CMD in the literature varies widely, likely due to differences in diagnostic modalities, each with varying sensitivities and specificities [11]. Additionally, advancements in diagnostic techniques over the years have likely improved diagnostic accuracy, whereas earlier studies may have used less sensitive methods, potentially underestimating the prevalence. Finally, the choice of CFR cut-off value, which can range between 2 and 2.5 depending on the study, also plays a significant role in influencing the diagnosis of CMD.

Understanding the prevalence of CMD in ANOCA/INOCA patients is crucial for better patient management. This systematic review aims to document the prevalence of CMD according to the diagnostic modalities used over time and to investigate how the choice of diagnostic cut-off values for CFR influences the reported prevalence of CMD in the identified studies.

## 2. Methods

This systematic literature review follows a prespecified research protocol registered on the international prospective register of systematic reviews (PROSPERO) (Evolution of Coronary Microvascular Dysfunction Prevalence Over Time and Across Diagnostic Modalities: A systematic Review; CRD42024579422). This study was conducted according to the Preferred Reporting Items for Systematic reviews and Meta-Analyses (PRISMA) criteria [12].

### 2.1. Literature Search and Selection Criteria

The systematic literature review was performed using the online databases PubMed, Embase, and The Cochrane Library, from inception to 1 May 2024. The following search terms were used: ((“coronary microvascular dysfunction”) OR (“coronary microvascular”) OR (“microvascular dysfunction”) OR (“coronary microvascular inflammation”) AND (prevalence)). The reference lists of included studies and relevant reviews were manually searched to identify additional relevant references.

### 2.2. Eligibility Criteria and Selection Process

Studies included in this review were written in English and had to fulfill the following criteria: (1) they were conducted on human subjects, (2) included participants aged ≥ 18 years, (3) reported the prevalence of CMD, and (4) utilized one of the following diagnostic methods: ICA, PET-CT, ETT, or CMR. Exclusion criteria included the following: (1) study design (conference abstracts, letters to the editor, reviews, meta-analyses, case reports, case series), (2) studies involving patients with obstructive coronary artery disease, (3) asymptomatic patients, and (4) recruitment period exceeding 20 years to minimize the impact of changes in diagnostic methods. In cases where the population overlapped, the study with the largest sample size or the most recent one was selected. Two reviewers independently identified the relevant studies (AZ and AS). In cases of disagreement, a third author (SF) made the final decision.

### 2.3. Quality Assessment and Data Extraction

The quality of the selected study was assessed using the Quality Assessment of Diagnostic Accuracy Studies tool (QUADAS-2) [13]. The QUADAS-2 tool is recommended for evaluating diagnostic accuracy in systematic reviews. It allows for the assessment of the risk of bias and the applicability of results in both noncomparative and comparative studies. The risk of bias is evaluated across four domains: patient selection, index test, reference standard, and flow and timing. The two reviewers independently extracted data, including the following information for each study: first author’s name, year of publication, sample size, prevalence of CMD, diagnostic modalities used for assessment, and diagnostic cut-off to define CMD.

### 2.4. Statistical Analyses

Univariate linear regression models were used to model the evolution of CMD prevalence over time by classifying the prevalence observed in each study according to the publication year. This analysis was repeated with the 2 different cut-off values for CFR (≤2 vs. ≤2.5) and for each diagnostic modality, individually (ICA, PET-CT, TTE, and CMR). If the slope of the regression was significantly different from zero (*p* < 0.05), a temporal trend was considered present. Analyses and figures were generated using GraphPad Prism 10.1.2 (GraphPad Software, Inc., La Jolla, CA, USA).

## 3. Results

### 3.1. Article Selection

**Figure 1:**
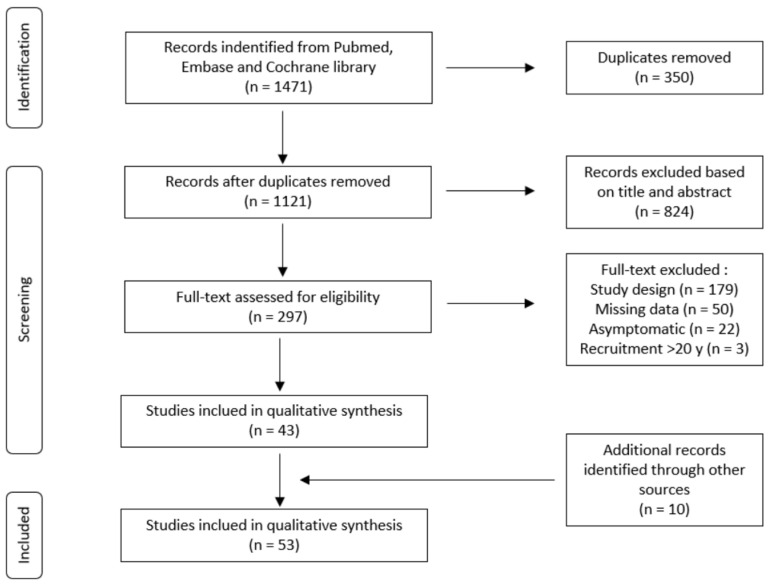
Flowchart diagram according to PRISMA criteria.

The flowchart is reported in Figure 1. Our initial search identified a total of 1471 articles, including 763 from PubMed, 679 from Embase, and 29 from The Cochrane Library. A total of 350 duplicates were identified. Of the remaining 1121 articles, 824 were excluded based on the title and abstract screening. Full-text analysis was performed on 297 studies, resulting in the identification of 43 articles eligible for inclusion [14,15,16,17,18,19,20,21,22,23,24,25,26,27,28,29,30,31,32,33,34,35,36,37,38,39,40,41,42,43,44,45,46,47,48,49,50,51,52,53,54,55,56]. Additionally, after reviewing the references of the included articles, 10 more were included [57,58,59,60,61,62,63,64,65,66]. Eventually, a total of 53 studies were included in this systematic review.

### 3.2. Quality Assessment

The risk of bias in the 53 included studies, assessed using the QUADAS-2 tool, is detailed in Table S1. Patient selection was the domain with the highest risk of bias, with 32 out of 53 studies identified as having a high risk of selection bias. The flow and timing domain followed, with 11 studies at high risk and 6 studies having unclear risk. In contrast, the index test and reference standard domains showed a low risk of bias across the included studies.

### 3.3. Study Characteristics and Prevalence of CMD

The 53 studies (published between 1998 and 2024) included a total of 16,602 patients with ANOCA/INOCA. CMD was assessed using ICA in 23 studies, using PET-CT in 15, using TTE in 8 and using CMR in 7. Regarding the studies based on ICA, 10 employed a Doppler guidewire [21,28,38,45,47,48,51,53,55,58], 11 used the bolus thermodilution [12,16,18,20,32,34,37,39,40,43,54], 1 used the continuous thermodilution [15], and 1 combined the use of Doppler guidewire and bolus thermodilution [19]. The sample sizes of the studies ranged from 11 to 2083 patients (Table 1). The reported prevalence of CMD across these studies showed significant variability, ranging from 22% to 100%.

**Figure 2:**
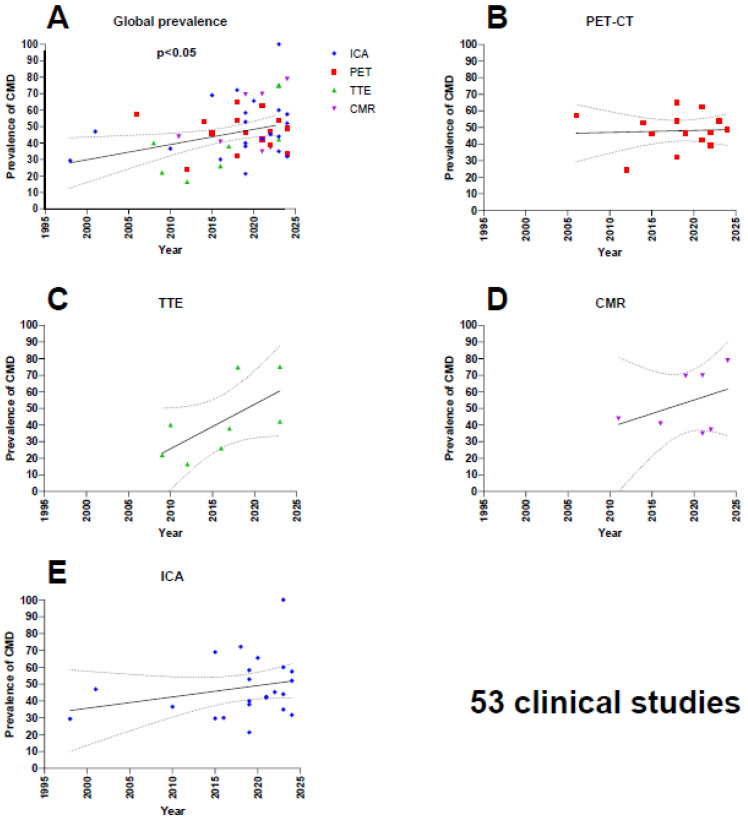
(**A**) Coronary microvascular resistance (CMD) prevalence over time with all diagnostic modalities. (**B**) CMD prevalence over time with positron emission tomography–computed tomography (PET-CT). (**C**) CMD prevalence with transthoracic echocardiogram (TTE). (**D**) CMD prevalence with cardiovascular magnetic resonance (CMR). (**E**) CMD prevalence with invasive coronary angiography (ICA).

Overall, a statistically significant increase in CMD prevalence over time across all diagnostic modalities was observed (*p* < 0.05, Figure 2). Depending on the diagnostic method, an increase in prevalence over time was observed for all modalities except for PET-CT, which showed a relatively stable prevalence over time of approximately 50% (*p* = 0.84). Notably, the prevalence rates from other diagnostic methods have been converging progressively towards the 50% prevalence of PET-CT.

### 3.4. The Prevalence According to the CFR Cut-Off (≤2.5 vs. ≤2)

Regarding the choice of the cut-off for CFR, 21 studies chose a CFR cut-off of ≤2.5 [14,17,21,23,25,30,37,38,42,44,47,49,50,55,56,57,58,60,61,62,65], 24 studies chose a cut-off of ≤2 [15,16,18,20,22,26,27,29,31,32,33,34,35,36,38,39,43,45,46,48,51,52,54,59], 4 studies used a different CFR cut-off [24,28,53,64], 2 studies used a qualitative cut-off [19,63], and 2 studies used only the index of microvascular resistance (IMR) [41,66].

**Figure 3:**
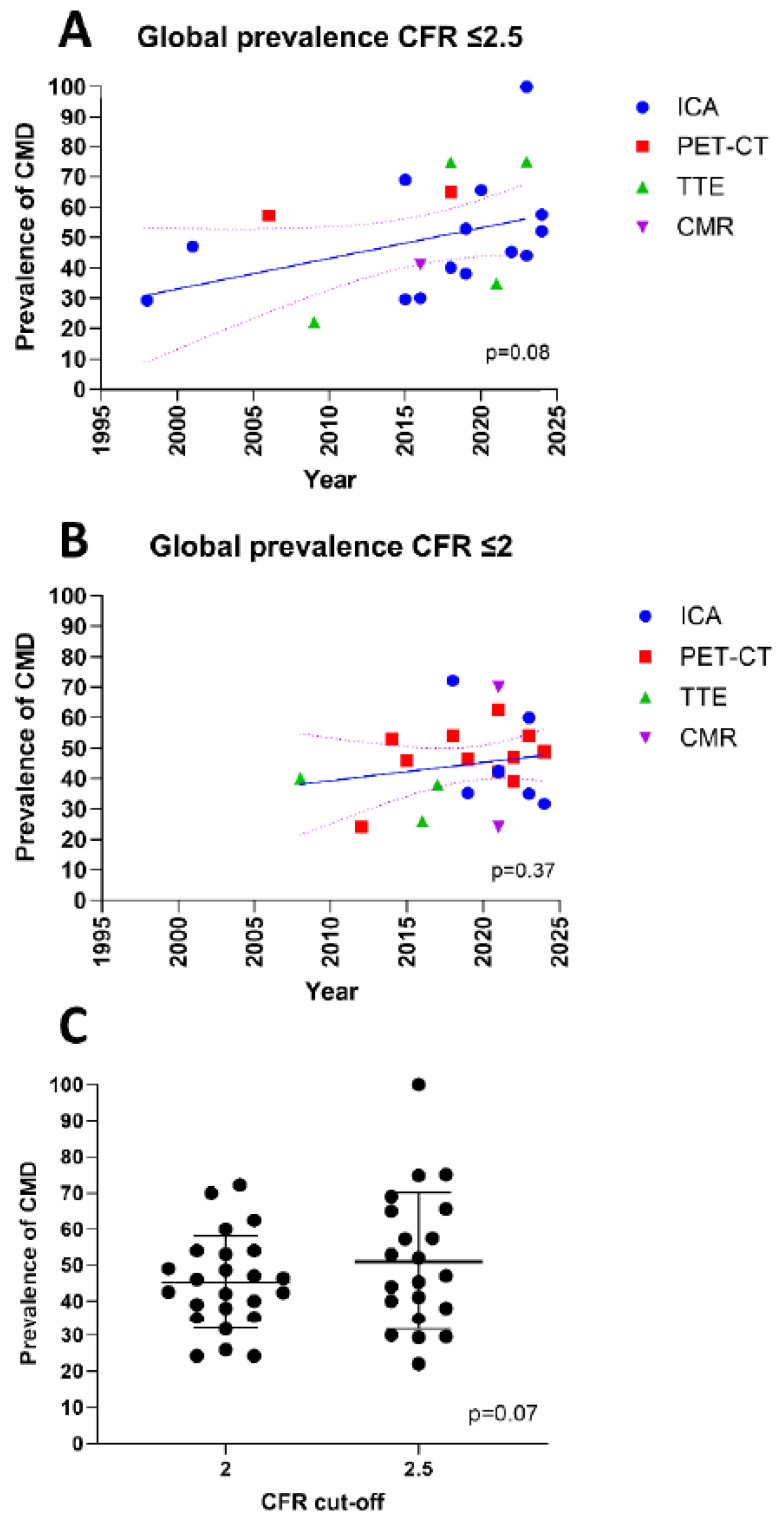
(**A**) Coronary microvascular resistance (CMD) prevalence with a cut-off ≤2.5. (**B**) CMD prevalence with a cut-off ≤2. (**C**) The prevalence according to the mean coronary flow reserve (CFR) cut-off.

The studies using a cut-off ≤2.5 were published between 1998 and 2024, whereas the studies using a cut-off ≤2 were published between 2008 and 2024. As expected, a trend toward a higher prevalence with a cut-off of ≤2.5 as compared to ≤2 was observed (50.1% and 45.1%, respectively, *p* = 0.07). Of interest, both cut-offs exhibited the same or similar trend to the one observed in general, with an increase in the prevalence of CMD over the years (*p* = 0.08 and *p* = 0.37, respectively, Figure 3).

## 4. Discussion

This systematic review revealed a significant increase in the prevalence of CMD reported in the literature from 1998 to 2024 (*p* < 0.05). When analyzing each modality individually, it becomes evident that the prevalence measured by TTE, CMR, and ICA has shown an upward trend, while for PET-CT, considered for a long time as the gold standard for non-invasive diagnosis, the prevalence has remained stable [67].

### 4.1. Impact of Diagnostic Modalities on CMD Prevalence over Time

This convergence towards a prevalence of approximately 50% can be attributed to significant advancements in both non-invasive and invasive diagnostic techniques, allowing for more detailed evaluation of coronary microcirculation over the years. For instance, TTE has seen notable improvements in spatial resolution, while CMR has benefited from enhanced spatial and temporal resolution in perfusion techniques as well as the development of protocols for better quantification of myocardial blood flow [68]. Similarly, coronary angiography has also evolved from using Doppler guides to assess blood flow [69] to the development of the thermodilution, initially with a bolus technique [70,71] and more recently with the continuous thermodilution technique, which is gradually being adopted and appears to be more precise [72,73,74,75]. However, in this systematic review, only one study utilized continuous thermodilution. It can be hypothesized that the prevalence of CMD measured using this method might be even higher due to its increased accuracy.

Moreover, the current increased interest in CMD (almost half of the studies in this systematic review were published in the last 4 years) may possibly be linked to a stronger belief that this diagnosis will be present in a patient, leading to particular attention being paid to the performance of the measurements and their interpretations, possibly carried out by expert centers and dedicated operators.

### 4.2. Comparative Advantage of Diagnostic Modalities

The criteria for CMD diagnosis are well established, relying on objective thresholds such as CFR ≤ 2.5 or elevated IMR values. However, the heterogeneity in CMD pathophysiology complicates its assessment, as structural and functional CMD can exhibit different diagnostic profiles. For instance, IMR values may remain normal in cases of functional CMD, despite significant microvascular dysfunction. Regarding the choice of diagnostic modalities, ICA provides a distinct advantage over other methods, allowing the measurement of microvascular resistance through the IMR, enabling a more detailed classification into CMD endotypes. Furthermore, for patients in whom VSA is also suspected, ICA allows for both conditions to be assessed in a single procedure, avoiding the need for multiple investigations. However, PET-CT has the significant advantage of measuring CFR across all three coronary territories, whereas ICA typically measures flow in only one territory. A recent study demonstrated that measuring flow in all three territories by ICA increases diagnostic accuracy in patients with ANOCA/INOCA compared to single-vessel testing [76].

### 4.3. Diagnostic Cut-Off Value for CFR

In the literature, it is well established that the cut-off value for CFR ≤ 2.5 is the current gold standard. However, many older studies have used a cut-off of 2. This shift in diagnostic cut-off values may be attributed to evolving recommendations, particularly with the publication of the European Association of Percutaneous Cardiovascular Intervention (EAPCI) expert consensus in 2020, which advocated for adopting a cut-off of ≤2 [1], whereas the recent European Society of Cardiology (ESC) guidelines for the management of chronic coronary syndrome recommend a cut-off of ≤2.5 [77]. When examining the mean prevalence of CMD based on the cut-off used, it is interesting to note that both cut-offs (CFR ≤ 2.5 and CFR ≤ 2.0) exhibit the same general trend, with an increase in the prevalence of CMD over the years (*p* = 0.08 and *p* = 0.37, respectively, as shown in Figure 3). While the EAPCI expert consensus advocates for a cut-off of ≤2, it is noteworthy that studies investigating the prognostic value of CFR are not unanimous in their findings. Studies that have shown a prognostic impact using a cut-off of ≤2.5 are primarily those based on invasive evaluation using Doppler guidewire [50]. In contrast, studies that have shown a prognostic impact with a cut-off of ≤2 have employed ICA thermodilution [78,79]. It is important to highlight that these studies not only included INOCA/ANOCA patients but also those who required revascularization. Our systematic literature review indicates that despite the increasingly frequent use of the ≤2 diagnostic cut-off, CMD prevalence reported in the literature has continued to rise over time. One could argue that this trend could be attributed to a potential increase in CMD, likely driven by the rising prevalence of cardiovascular risk factors in the population [80]. However, the stable prevalence reported in studies using PET-CT suggests that advancements in diagnostic methods are more likely responsible for the observed increase in reported prevalence over time. Future research should focus on assessing the prognostic value of these cut-offs, particularly in INOCA/ANOCA patients, using current gold-standard diagnostic methods such as PET-CT for non-invasive assessment or ICA for invasive evaluation.

## 5. Limitations

The primary limitation of this systematic review is related to the risk of bias, particularly regarding patient selection. There was a considerable degree of variability in the inclusion and exclusion criteria across the selected studies. Notably, the definition of obstructive coronary artery disease varied significantly between studies. Additionally, five studies [53,54,55,63,65] only included women, which also contributes to a selection bias. Furthermore, the only study that included patients with atrial fibrillation reported a 100% prevalence of CMD, although this study had the smallest sample size (n = 11). Finally, it is important to note that the prognostic implications of CFR extend beyond CMD and include other conditions such as dilative and hypertrophic cardiomyopathies [81].

### Future Perspective

This literature review opens several future perspectives. First, determining a gold-standard diagnostic modality for CMD is inherently complex. Currently, several diagnostic modalities—PET-CT, ICA, TTE, and CMR—are used, often being compared against each other. However, a definitive study involving a single cohort of patients undergoing all of these modalities simultaneously is still lacking.

Invasive methods with CFR or IMR measurements are often considered the gold standard due to the theoretical precision allowed by their invasive aspect, but the fact that these methods are invasive limits their applicability in broader clinical practice. Second, regarding the invasive diagnosis of CMD, two methods are currently employed: bolus thermodilution and continuous thermodilution. Further studies are needed to determine which method provides the best sensitivity, specificity, and reproducibility. Third, the impact on prevalence when CMD is investigated in all of the coronary territories compared to a single one needs to be clarified. Additionally, the impact of CMD on morbidity, quality of life, and healthcare costs, as highlighted by a previous study, is significant. Thus, given the high prevalence of CMD in patients with angina and non-obstructive coronary arteries, a systematic evaluation of microcirculation in these patients might be routinely considered. Finally, future research must explore the effectiveness of current state-of-the-art treatments and their impact on coronary microcirculation.

## 6. Conclusions

The prevalence of CMD in patients with non-obstructive coronary artery disease has been debated for a long time, with reported rates showing significant variation. However, the current data suggest that recent studies increasingly converge towards a prevalence of around 50%, regardless of the diagnostic method used—a figure that has been consistently observed in PET-CT assessments since the earliest studies. Given the well-established association between CMD and an elevated risk of major adverse cardiac events, systematically evaluating coronary physiology in INOCA/ANOCA patients during coronary angiography may lead to improved outcomes. Such an approach could facilitate earlier diagnosis and the implementation of targeted treatments aimed at reducing MACE risk.

## Figures and Tables

**Table 1:** Sample Size, prevalence, number of positive patients, diagnostic methods used, and cut-off diagnostic used for the selected study.

Study	Sample Size	Prevalence CMD (%)	No. Positive	Diagnostic Modality	Cut-Off
Zornitzki [14], 2024	245	57.5	141	ICA (themodilution bolus)	CFR < 2.5
Souza [15], 2024	400	49	196	PET-CT	CFR < 2
Patel [16], 2024	1425	48.6	692	PET-CT	MBFR < 2
Paolisso [17], 2024	56	52	29	ICA (thermodilution continuous)	CFR < 2.5
Niewiara [18], 2024	101	31.7	32	ICA (themodilution bolus)	CFR < 2
Kong [19], 2024	91	79	72	CMR	Perfusion defect
Zaragoza [20], 2023	60	60	36	ICA (themodilution bolus)	CFR < 2
Vink [21], 2023	1007	44	443	ICA (Doppler guidewire and thermodilution bolus)	CFR ≤ 2.5
Vaz Ferreira [22], 2023	20	35	7	ICA (themodilution bolus)	CFR ≤ 2
Pintea Bentea [23], 2023	11	100	11	ICA (Doppler guidewire)	CFR < 2.5
Kim [24], 2023	202	42	84	Doppler TTE	CFvR < 2.3
Erhardsson [25], 2023	202	75	151	Doppler TTE	CFR < 2.5
Bhandiwad [26], 2023	239	54	130	PET-CT	MFR < 2
Weber [27], 2022	174	47	81	PET-CT	MFR < 2
Slivnick [28], 2022	99	34.3	34	CMR	MPRI < 1.51
Lopez [29], 2022	249	39	98	PET-CT	CFR < 2
Lee [30], 2022	287	45,3	130	ICA (Doppler guidewire)	CFR ≤ 2.5
Arnold [31], 2021	101	70	70	CMR	MPR < 2
Weber [32], 2021	111	42.3	47	PET-CT	MFR < 2
Schumann [33], 2021	66	24.2	16	CMR	MPR < 2
Ozcan [34], 2021	80	42.5	34	ICA (thermodilution bolus)	CFR < 2
Liao [35], 2021	514	62.5	321	PET-CT	CFR < 2
Jansen [36], 2021	252	42	107	ICA (thermodilution bolus)	CFR < 2
Kumar [56], 2020	163	65.6	107	ICA (thermodilution bolus)	CFR < 2.5
Rahman [57], 2019	85	52.9	45	ICA (Doppler guidewire)	CFR < 2.5
Vita [38], 2019	886	46.4	411	PET-CT	CFR < 2
Suda [39], 2019	187	58.3	109	ICA (thermodilution bolus)	CFR < 2
Sara [40], 2019	129	38	49	ICA (Doppler guidewire)	CFR < 2.5
Kotecha [66], 2019	23	69.6	16	CMR	IMR ≥ 25
Pargaonkar [41], 2019	150	21.3	32	ICA (thermodilution bolus)	IMR ≥ 25
Anderson [42], 2019	222	40	89	ICA (thermodilution bolus)	CFR ≤ 2.5
Safdar [37], 2018	124	65	81	PET-CT	CFR < 2.5
Taqueti [43], 2018	201	54	108	PET-CT	CFR < 2
Shah [44], 2018	202	74.8	151	Doppler TTE	CFR < 2.5
Schroder [65], 2018	97	32	31	PET-CT	MBFR < 2.5
Ford [45], 2018	151	72.2	109	ICA (thermodilution bolus)	CFR < 2
Nel [46], 2017	183	38	70	Doppler TTE	MBFR < 2
Sara [47], 2016	926	30	281	ICA (Doppler guidewire)	CFR ≤ 2.5
Kato [58], 2016	56	41	23	CMR	CFR < 2.5
Mygind [48], 2016	919	26.2	241	Doppler TTE	CFVR < 2
Valenzuela-Garcia [49], 2015	314	29.6	93	ICA (Doppler guidewire)	CFR < 2.5
Sara [50], 2015	1439	69	998	ICA (Doppler guidewire)	CFR ≤ 2.5
Taqueti [51], 2015	761	46	349	PET-CT	CFR < 2
Murthy [52], 2014	1218	53	641	PET-CT	CFR < 2
Sakamoto [64], 2012	73	16.4	12	Doppler TTE	CFR ≤ 2.8
Srivaratharajah [59], 2012	376	24,2	91	PET-CT	MBFR < 2
Ishimori [63], 2011	18	44	8	CMR	Perfusion defect
Pepine [53], 2010	153	36,6	56	ICA (Doppler guidewire)	CFR < 2.32
Sicari [61], 2009	394	22	87	Doppler TTE	CFR < 2.5
Sade [54], 2008	65	40	26	Doppler TTE	CFR < 2
Graf [61], 2006	89	57.3	51	PET-CT	CFR < 2.5
Reis [55], 2001	159	47	74	ICA (Doppler guidewire)	CFR < 2.5
Hasdai [60], 1998	203	29.3	59	ICA (Doppler guidewire)	CFR < 2.5

ICA: invasive coronary angiography; CFR: coronary flow reserve; PET-CT: positron emission tomography–computed tomography; MBFR: myocardial blood flow reserve; CMR: cardiac magnetic resonance; TTE: transthoracic echocardiography; CFvR: coronary flow velocity reserve.

## Supplementary Materials

The following supporting information can be downloaded at https://www.mdpi.com/article/10.3390/jcm14030829/s1, Table S1: Quality assessment, risk of bias of the studies included.

## Abbreviations

CMD	Coronary microvascular dysfunction
ANOCA	Angor with non-obstructive coronary arteries
INOCA	Ischemia with non-obstructive coronary arteries
ICA	Invasive coronary angiography
PET-CT	Positron emission tomography–computed tomography
TTE	Transthoracic echocardiography
CMR	Cardiac magnetic resonance
CFR	Coronary flow reserve
