# Supplementary material for: Evolution of Coronary Microvascular Dysfunction Prevalence over Time and Across Diagnostic Modalities in Patients with ANOCA: A Systematic Review
Source: J Clin Med. 2025 Jan 27;14(3):829. doi: 10.3390/jcm14030829 (PMC11818762; doi:10.3390/jcm14030829)
Supplement: Supplementary file 1 [file jcm-14-00829-s001.zip › jcm-3377783-supplementary.pdf]

# Supplementary Material

**Supplemental Table S1. Quality assessment, risk of bias of the studies included.**

RISK OF BIAIS

| Study | Patient selection | Index Test | Reference Standard | Flow and Timing |
|---|---|---|---|---|
| Zornitzki, 2024 | | | | |
| Souza, 2024 | | | | |
| Patel, 2024 | | | | |
| Paolisso, 2024 | | | | |
| Niewiara, 2024 | | | | |
| Kong, 2024 | | | | |
| Zaragoza, 2023 | | | | |
| Vink, 2023 | | | | |
| Vaz Ferreira, 2023 | | | | |
| Pintea Bentea, 2023 | | | | |
| Kim, 2023 | | | | |
| Erhardsson, 2023 | | | | |
| Bhandiwad, 2023 | | | | |
| Weber, 2022 | | | | |
| Slivnick, 2022 | | | | |
| Lopez, 2022 | | | | |
| Lee, 2022 | | | | |
| Arnold, 2021 | | | | |
| Weber, 2021 | | | | |
| Schumann, 2021 | | | | |
| Ozcan, 2021 | | | | |
| Liao, 2021 | | | | |
| Jansen, 2021 | | | | |
| Kumar, 2020 | | | | |
| Rahman, 2019 | | | | |
| Vita, 2019 | | | | |
| Suda, 2019 | | | | |
| Sara, 2019 | | | | |
| Kotecha, 2019 | | | | |
| Pargaonkar, 2019 | | | | |
| Anderson, 2019 | | | | |
| Safdar, 2018 | | | | |
| Taqueti, 2018 | | | | |
| Shah, 2018 | | | | |
| Schroder, 2018 | | | | |
| Ford, 2018 | | | | |
| Nel, 2017 | | | | |
| Sara, 2016 | | | | |
| Kato, 2016 | | | | |

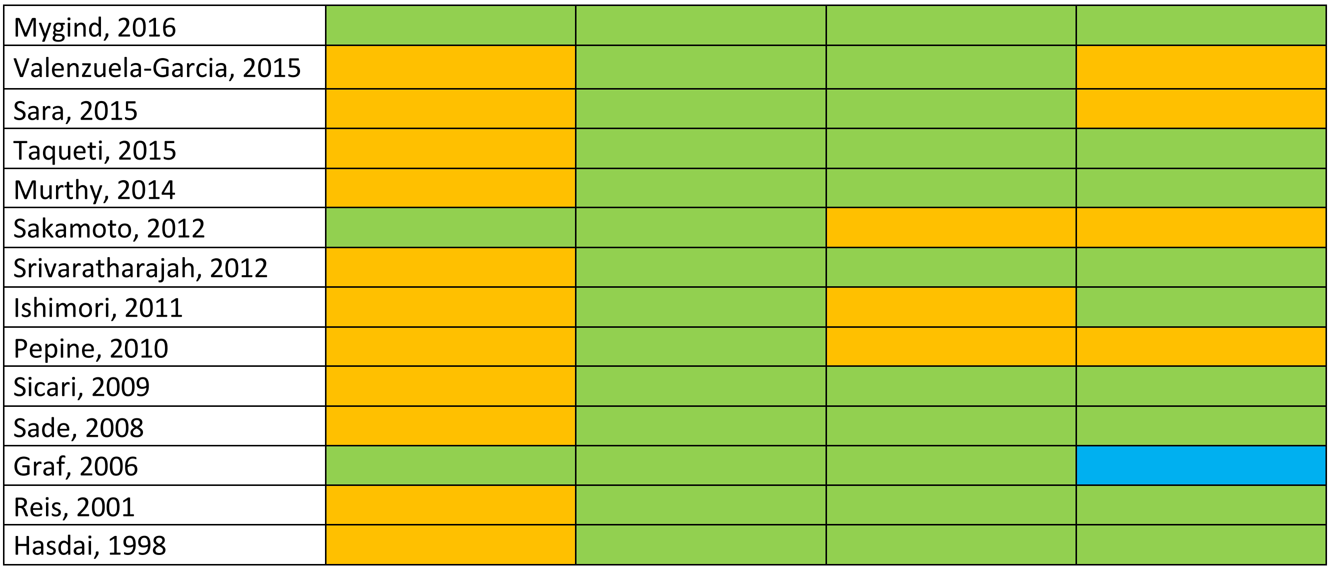


**Green : low risk**

**Orange : high risk**

**Blue : unclear**
